# The Gut–Liver Axis in MASLD: From Host–Microbiome Crosstalk to Precision Therapeutics

**DOI:** 10.3390/microorganisms14020471

**Published:** 2026-02-14

**Authors:** Ji Zhou, Bowen Zhu, Ziqian Bing, Tingting Wang, Yue Zhao

**Affiliations:** 1 The State Key Laboratory of Pharmaceutical Biotechnology, Chemistry and Biomedicine Innovation Center (ChemBIC), Division of Immunology, Medical School, Nanjing University, Nanjing 210093, China; 221230036@smail.nju.edu.cn (J.Z.); 221230038@smail.nju.edu.cn (B.Z.); mg21350022@smail.nju.edu.cn (Z.B.); 2 Jiangsu Key Laboratory of Molecular Medicine, Division of Immunology, Medical School, Nanjing University, Nanjing 210093, China

**Keywords:** gut–liver axis, MASLD, gut microbiome, dysbiosis, microbiome-based therapeutics

## Abstract

Metabolic dysfunction-associated steatotic liver disease (MASLD) is an emerging global health challenge with limited effective therapeutic options. The gut microbiota, at the interface of host metabolism and immunity, acts as a critical disease modifier via the gut–liver axis. This review goes beyond cataloging its associations and synthesizes how intrinsic and extrinsic factors sculpt a permissive microbial ecosystem. These factors likely converge to establish a state of “metabolic dysbiosis”, fueling MASLD progression through three core mechanisms: compromised intestinal barrier integrity with immune activation, dysregulation of key microbial metabolite axes, and direct hepatic insult from gut-derived products. Next, we evaluate the translational landscape through a mechanism-informed precision framework, with an emphasis on how microbiome-based interventions could be aligned with non-invasive biomarkers increasingly used for MASLD risk stratification and treatment monitoring. By integrating evidence across scales, this review aims to frame a roadmap from microbiome correlations to causality-driven, personalized therapeutic strategies for MASLD.

## 1. Introduction

Metabolic dysfunction-associated steatotic liver disease (MASLD), previously referred to as “non-alcoholic fatty liver disease” (NAFLD), has become the most common chronic liver disease in the world [1,2]. Its clinical course gradually develops from simple liver steatosis to metabolic dysfunction-associated fatty hepatitis (MASH) and can further evolve into cirrhosis and hepatocellular carcinoma (HCC). In recent years, with an increasing prevalence of metabolic abnormalities such as obesity and type 2 diabetes, the incidence of MASLD and its potentially serious health hazards has surged [3]. This emerging health crisis thus imposes a great public health burden due to its association with high risks of cardiovascular events, cancer, type 2 diabetes, chronic kidney disease progression, and mortality [4].

Due to this disease’s insidious and asymptomatic nature, some MASLD patients present with irreversible advanced fibrosis or even HCC at the time of initial diagnosis [5]. Early detection is therefore of prime importance. However, in clinical practice, the current gold standard for MASLD diagnosis, liver biopsy, is an invasive method [6]. In addition, non-specific symptoms such as hepatitis often lead to misdiagnosis [7]. Therefore, there is an urgent need for effective, sensitive, and repeatable non-invasive biomarkers. Emerging evidence suggests that microbiota-associated signatures derived from stool, blood, or microbial metabolites may complement existing non-invasive tools (e.g., Controlled Attenuation Parameter, Magnetic Resonance Imaging - Proton Density Fat Fraction, fibrosis scores) for MASLD risk stratification and longitudinal monitoring [2,8,9]. Regarding treatment, poor patient adherence frequently leads to disappointing outcomes of lifestyle interventions, while approved drug treatment regimens are very limited [10]. This has formed a therapeutic impasse that refocuses on the gut microbiome. An increasing number of clinical trials on microbiota modulation now predict this as a likely future pillar of personalized MASLD management [11].

MASLD is a multi-system metabolic dysbiosis characterized by insulin resistance and metabolic syndrome [12]. Its pathogenesis is “multiple-hit” and includes liver lipid accumulation, inflammatory cascade reaction activation, intestinal microbiome dysbiosis, and genetic regulation [13]. The intestinal microbiome, containing trillions of bacteria-dominated microorganisms, as well as archaea, fungi, and viruses, is one of the key participants [13]. In human studies, MASLD-associated dysbiosis has been reported most consistently in adult cohorts enriched for obesity and/or type 2 diabetes, conditions that are closely intertwined with (and can confound) microbiome signatures attributed to MASLD itself [14]. Across such cohorts, reduced microbial diversity and enrichment of pathobiont-associated taxa (often including *Proteobacteria*/*Enterobacteriaceae*) together with depletion of fiber-associated genera (e.g., *Bifidobacterium* and *Akkermansia*) are recurrent observations, although the direction and magnitude of these shifts vary with metabolic background and study design [14]. In addition, a recent high-resolution multi-omics study reported trans-kingdom dysbiosis in a large, single-sex population (female nurses; 211 MASLD cases and 502 controls), identifying shifts in 66 bacterial species alongside viral taxa perturbations [15].

The gut and the liver display multidimensional interdependence: embryologically, the liver develops from the foregut; anatomically, it receives portal blood rich in microbial and metabolic products from the gut while secreting bile into the intestinal lumen. This bidirectional crosstalk has been termed the gut–liver axis [13]; through this axis, the gut microbiota has profound impacts on hepatic health. Despite progress, this field still faces numerous limitations that require critical interpretation of evidence. First, many findings only demonstrate correlations, necessitating further human studies to establish causality. Second, current trials primarily rely on animal models, the transferability of which to human models often remains uncertain. Third, existing research predominantly focuses on bacteria, with limited exploration of fungi, viruses, and archaea within the gut ecosystem [11]. This review will highlight these limitations and emphasize their critical importance in future research agendas.

This paper explores these lacunae by adopting a mechanistic and integrative perspective. First, we analyze how host factors program a MASLD-permissive microbiome. Then, we dissect the core causal pathways linking dysbiosis to hepatic pathology. Finally, we critically review therapeutic approaches by mapping them onto these mechanistic pillars, with the aim of accelerating the development of targeted, microbiome-informed precision medicine for MASLD.

## 2. Host and Behavioral Determinants of a MASLD-Permissive Microbiome

Gut microbiome composition and function are in continuous flux due to a complex interaction between host genetics and a multitude of modifiable lifestyle factors. The section deconstructs how these determinants interact at multiple levels to establish a dysbiotic state that either predisposes to or exacerbates MASLD.

### 2.1. Sex

Sex has a profound impact on gut microbiota composition and MASLD susceptibility. The disease is more prevalent in males [16,17,18]. The protective role of premenopausal estrogen is a central hypothesis. Indeed, premenopausal women have been observed to harbor a more abundant and metabolically favorable gut microbiota than their male counterparts [19,20,21,22].

The microbiota associated with MASLD exhibit sex-specific distributions. For instance, the genera *Christensenella* and *Limosilactobacillus* are more frequent in women, while *Beduinibacterium* and *Anaerotruncus* are more common in men [23]. Compared with healthy controls, male MASLD patients typically demonstrate reduced gut microbial α-diversity and distinct compositional shifts. In contrast, studies in female patients showed relatively higher α-diversity and opposite flora composition characteristics [24].

Animal models support these observations. When fed a high-fat diet, male mice exhibit more severe metabolic dysfunction, including increased fasting glucose, insulin, and alanine aminotransferase (ALT) levels [25]. The functional profiles of gut-derived metabolites also diverge between the sexes. A particularly important phenomenon observed in males is a change in the susceptibility of the liver to microbiota-derived inflammatory signaling, exemplified by increased hepatic expression of Toll-like receptor 4 (TLR4) and parallel elevations in levels of Gram-negative bacterial lipopolysaccharide (LPS) [18]. Thus, sex needs to be a required covariate for all gut microbiota analyses. Research into sex-specific differences in MASLD patients’ gut microbiota is essential. More elaborate knowledge of these dimorphic profiles and their disease-specific implications will prove essential to the development of sex-specific diagnostic and therapeutic approaches.

### 2.2. Genetics

MASLD pathogenesis arises from a complex interplay involving host genetics, epigenetics, and the gut microbiome. Family and twin studies confirm that the disease has a heritable component [26,27,28,29,30]. Twin cohort analyses in humans show that monozygotic twins have more similar microbiome profiles than dizygotic twins, supporting a heritability/host-genetic contribution to microbiome variation (observational human evidence) [31]. On the other hand, microbial metabolites have been shown (mainly in mechanistic preclinical models) to modulate host signaling/epigenetic regulation and may thereby contribute to disease progression, but direct causal evidence in humans remains limited [32,33].

Specific genetic variants modulate MASLD risk through interactions with the gut microbiome. For example, in obese youth, fecal abundances of *Gemmiger* and *Oscillospira* combined with the patatin-like phospholipase domain-containing protein 3 (*PNPLA3*) rs738409 risk variant predicted liver fat content [34]. Another study found that the *PNPLA3* rs738409 G/G genotype is associated with reduced *Desulfobacteraceae* but increased fungal genera, including *Candida* and *Aspergillus* [35]. A novel mechanistic link was provided by preclinical genetic evidence (animal models) showing that intestinal *TM6SF2* perturbation can worsen MASLD/MASH phenotypes alongside microbiome shifts, consistent with a gut–liver axis mechanism [36].

Collectively, these findings underscore the existence of dynamic gene–microbiome–metabolite axes in MASLD. Future research should employ integrated multi-omics approaches to delineate these complex interactions. Such mechanistic insights will be essential for developing genotype-directed, microbiome-informed therapeutic strategies.

### 2.3. Diet

Diet patterns are a major modifiable determinant of the intestinal microbiome and are strongly associated with MASLD risk/severity. However, much of the mechanistic evidence comes from preclinical studies, while human data are often observational [37,38,39,40,41]. Western-style dietary patterns have been shown to reshape gut microbiome composition and function across multiple study types, often shifting communities toward lower fiber-associated fermentation capacity and altered metabolite output [42]. In this framework, reduced short-chain fatty acid (SCFA) production and impaired barrier support represent plausible downstream consequences of low microbiota-accessible carbohydrate intake, providing a mechanistic link to metabolic dysfunction relevant to MASLD rather than a uniform, deterministic sequence in all individuals [42,43,44].

On the contrary, the Mediterranean diet can promote the benign transformation of the intestinal flora. These diet-induced bacterial flora changes can affect the host’s lipid/glucose homeostasis, bile acid metabolism, and immune function [45,46].

By analyzing specific micronutrients, the following mechanism is revealed: fiber-rich whole grains can reduce inflammation and improve barrier function [47]. A diet high in saturated fats can promote the growth of pro-inflammatory bacteria [48,49], while unsaturated fatty acids can enrich *Bifidobacterium* and butyrate-producing bacteria [50]. Emulsifiers and some food additives have been shown to alter the human gut microbiome in interventional settings (e.g., in a randomized controlled feeding study of carboxymethylcellulose in healthy adults), while other evidence comes from ex vivo human microbiota experiments and preclinical studies; therefore, their role in metabolic dysfunction should be described as supported but context-dependent rather than universally causal [51,52,53].

Therefore, dietary intervention has always been the basic and cost-effective core strategy of MASLD management, and targeted dietary adjustment provides a feasible path for restoring microbial and metabolic health.

### 2.4. Circadian Rhythm

A regular circadian rhythm is characterized by interconnected diurnal rhythms of the host circadian clock, liver metabolism, and gut microbiota [54]. This relationship is bidirectional: the host clock drives microbial rhythms by entrainment via feeding–fasting cycles [55,56,57,58], whereas bacterial metabolites, in turn, drive host transcriptional and metabolic oscillations [59]. Disruption of this harmony—as evidenced by high-fat diet (HFD) or aberrant light cycles—blunts microbial rhythms and is a proposed risk factor for MASLD.

In this context, time-restricted eating (TRE) shows metabolic benefits in preclinical models and has emerging interventional human evidence in MASLD; for example, one study reports a 4-week TRE regimen in 19 MASLD participants (human intervention) alongside mechanistic mouse experiments. Claims should thus be framed as supported by interventional evidence but still developing [60,61]. Interestingly, with regard to the beneficial effects of exercise, their magnitude may also depend on the timing of exercise training, as late-day exercise proves to be noticeably more effective in MASLD improvement, especially in reducing hepatic lipid accumulation, than early-day exercise [62].

### 2.5. Smoking

Smoking is common in patients with liver disease and is also independently associated with MASLD risk after adjusting for confounders [63]. Tobacco exposes the intestines to toxic compounds such as nicotine and aldehydes. Smoking is epidemiologically associated with microbiome alterations and MASLD risk, while mechanistic links involving barrier dysfunction and hepatic lipid accumulation are supported mainly by preclinical studies; thus, these effects should be described as potential pathways rather than established causation in humans [64,65].

Nicotine is an extensively studied alkaloid which, in combination with a high-fat diet, can have a harmful impact on the occurrence and progression of MASLD by upregulating the inflammatory response, increasing oxidative stress, promoting abdominal lipolysis, and improving liver fat synthesis [66]. Observational data show that smoking can lead to intestinal flora dysbiosis, which is usually characterized by changes in the proportion of *Firmicutes*/*Bacteroidetes*, an increase in *Proteobacteria*, and a decrease in beneficial genera such as *Lactobacillus* and *Bifidobacterium*. This may cause changes in the metabolite spectrum, including abnormal levels of ceramides, ethanol, LPS, and other substances [67,68]. Therefore, smoking may have a negative impact on MASLD progression through the lung–gut–liver axis, mediated by microbial metabolites. A key mechanism involves intestinal nicotine accumulation, which activates AMP-activated protein kinase α (AMPKα); this, in turn, stabilizes sphingomyelin phosphodiesterase 3 (SMPD3), increasing ceramide levels and driving MASH progression in nicotine-exposed mouse models [69].

Conversely, the gut microbiome can mitigate the damage. *Bacteroides xylanisolvens* degrades nicotine via the NicX enzyme, which decreases liver injury [69]. This exemplifies a complex lung–gut–liver axis in which microbial metabolism may modify environmental exposures.

### 2.6. Drugs

In addition to antibiotics, common drugs such as proton pump inhibitors (PPIs) and metformin have also been proven to have significant effects on the intestinal microbiome [70]. Among them, PPI use is observationally associated with reduced microbial diversity and compositional shifts [71]. Early PPI use may lead to longer flora dysbiosis and increase susceptibility to obesity [72]. Metformin treatment has been reported to alter gut microbiome composition in interventional human studies, and these changes may contribute to metabolic effects, though causal mediation in MASLD specifically remains to be fully established [73,74]. Studies suggest that this flora change is related to its metabolic improvement. Although the link between intestinal flora dysbiosis and MASLD pathogenesis has been confirmed, the question how commonly used prescription drugs affect the course of MASLD by regulating the microbiome is still a direction of insufficient research but with broad prospects.

**Figure 1:**
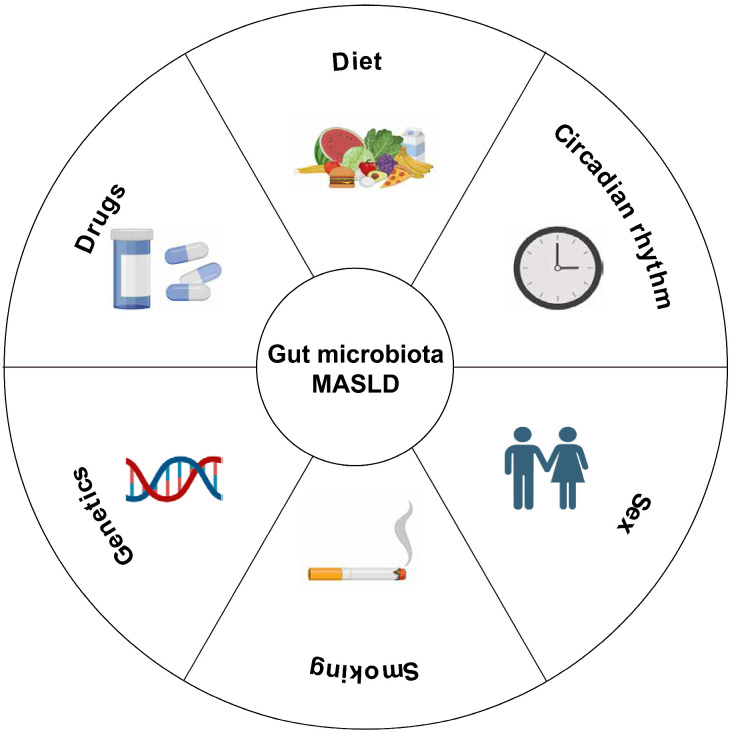
Host and behavioral factors affecting the MASLD gut–liver axis. This diagram explains the interaction between host endogenous and behavioral factors in shaping the composition and function of intestinal microorganisms. These interactions regulate the gut–liver axis and affect the occurrence and development of MASLD. MASLD: metabolic dysfunction-associated fatty liver disease.

There is a two-way interaction between drugs and the microbiome. Intestinal microorganisms can change the pharmacological properties of drugs, thus affecting their bioavailability, activity, and toxicity. These microbial-mediated drugs can modify individualized therapeutic responses, highlighting the important role of the microbiome in regulating drug safety and effectiveness (Figure 1).

## 3. Core Mechanisms: From Microbial Dysbiosis to Hepatic Pathology

**Figure 2:**
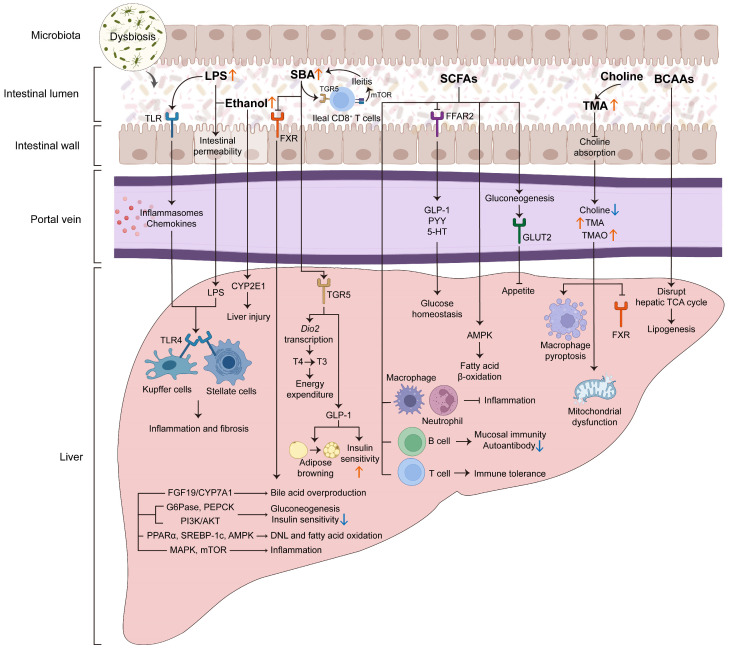
The mechanism by which intestinal flora dysbiosis promotes MASLD progression through the gut–liver axis. Intestinal flora dysbiosis can participate in MASLD occurrence and development through multiple interrelated pathways. (A) Intestinal barrier damage and immune activation: Flora dysbiosis can destroy the close connection and increase intestinal permeability. This causes harmful substances such as LPS to be displaced into the portal circulation. In the liver, these molecules can activate TLRs on Kupffer cells and hepatic stellate cells, trigger NF-κB-mediated inflammatory reactions, and promote fibrosis. (B) Microbial metabolite dysregulation: Bile acid metabolism can affect the signaling pathways of FXR and TGR5, thereby regulating glucose homeostasis, lipid metabolism, and the inflammatory response. Short-chain fatty acids regulate lipid oxidation in the liver by regulating the secretion of GLP-1, PYY, and 5-HT, Treg cell differentiation, and macrophage polarization; choline deficiency will lead to an increase in TMA/TMAO, thus inhibiting the FXR signaling pathway and promoting mitochondrial dysfunction, macrophage death, and fibrosis; finally, intestinal ethanol and branched-chain amino acid metabolites can aggravate liver steatosis, inflammation, and mitochondrial dysfunction. LPS: lipopolysaccharide; TLRs: toll-like receptors; NF-κB: nuclear factor kappa-light-chain-enhancer of activated B cells; FXR: farnesoid X receptor; TGR5: Takeda G protein-coupled receptor 5; GLP-1: glucagon-like peptide-1; PYY: peptide YY; 5-HT: serotonin; TMA: trimethylamine; TMAO: trimethylamine N-oxide.

Dysbiosis in MASLD is not merely a compositional shift but a functional alteration that disrupts key host pathways. We focus on three interconnected mechanistic axes that represent convergent functional pathways frequently implicated in MASLD while acknowledging that the relative contribution of each axis differs substantially across patient subgroups (Figure 2).

### 3.1. The Barrier–Immune Axis: From Leaky Gut to Hepatic Inflammation

The intestinal barrier is composed of a mechanical barrier, a chemical barrier, an immune barrier, and a biological barrier. It is an important line of defense that limits the entry of harmful pathogens and pathogenic factors into the blood circulation of the gastrointestinal tract [75]. Research based largely on animal models indicates that intestinal bacterial overgrowth and barrier dysfunction are key pathogenic factors, and their severity is positively linked to steatohepatitis progression in experimental settings [76,77].

In both animal models and selected human studies, an imbalanced intestinal microbiota has been associated with impaired tight junction integrity, facilitating translocation of LPS and other bacterial products into the portal circulation, a phenomenon most prominently observed in patients with advanced steatohepatitis rather than isolated steatosis [78,79]. As one of the most important pathogen-associated molecular patterns (PAMPs), LPS targets TLR4 on the surface of Kupffer cells and hepatic stellate cells, thus triggering NF-κB-mediated inflammation and fibrosis signaling pathways [80,81,82,83,84]. This inflammatory process will further destroy the integrity of the intestinal mucosa and form a vicious cycle that aggravates the entry of toxic substances.

At the molecular level, the PAMPs and damage-associated molecular patterns (DAMPs) produced by the intestine induce the formation of NOD-like receptor family pyrin domain containing 3 (NLRP3) inflammasome by activating TLR, leading to the activation of cysteine-aspartic acid protease-1 (caspase-1), cell death, and pro-inflammatory cytokine secretion, specifically of IL-1β and IL-18. These inflammatory mediators promote inflammatory cell infiltration, thus aggravating liver damage [85,86,87,88]. In addition, animal experimental data demonstrate that high fat concentration will enhance the palmitoylation of myeloid differentiation primary response 88 (MyD88), which, in turn, enhances TLR/MyD88 pathway activity and ultimately aggravates the inflammatory response of the liver and abnormal lipid metabolism [89,90]. Collectively, these findings show how an imbalance of the microbiota—namely, MASLD-associated gut dysbiosis—triggers the gut–liver axis, leading to the maintenance of liver inflammation, insulin resistance, and fibrosis.

While the detailed molecular cascade linking intestinal barrier dysfunction to hepatic inflammation has been well elucidated in animal models, emerging human studies provide key clinical validation for this axis. Patient data show that gut microbiota dysregulation—such as increased bile salt hydrolase activity—is associated with decreased tight junction protein expression and elevated plasma endotoxin levels, in addition to being positively correlated with the degree of liver fibrosis [91,92]. Intervention studies further demonstrate that modulating microbiota function through small-molecule inhibitors or metabolites (e.g., propionate) can improve the intestinal barrier and reduce systemic inflammation, partly involving the TLR/NF-κB pathway [91,93]. Portal circulation studies directly confirm the translocation of microbial products and their association with hepatic inflammatory activation [92]. Nevertheless, finer molecular mechanisms, such as NLRP3 inflammasome activation and MyD88 palmitoylation, remain primarily elucidated through animal models. Future human studies integrating direct intestinal permeability measurements with multi-omics analyses are needed to fully validate the clinical translational value of this axis as a therapeutic target.

**Figure 3:**
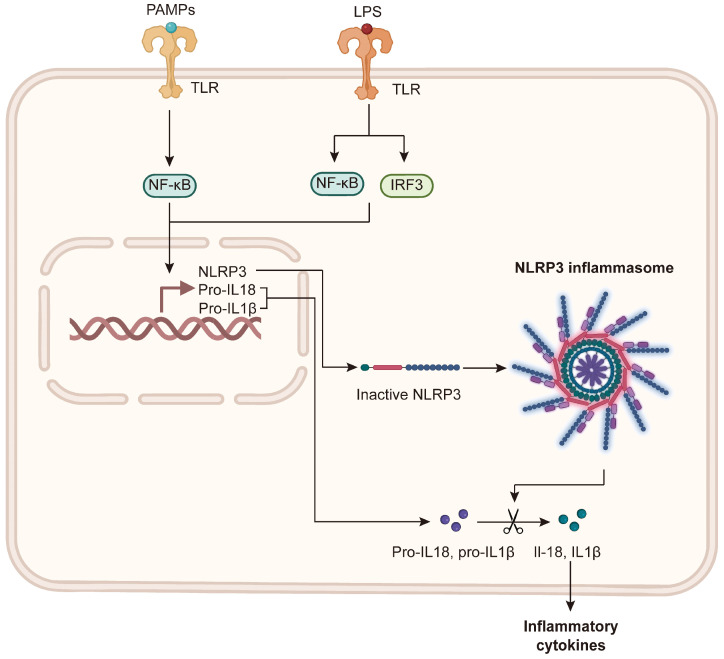
Molecular mechanisms of gut-derived PAMP-induced hepatic inflammation. Translocated gut-derived harmful substances, such as LPS, act as PAMPs. These are recognized by PRRs, including TLR4 and TLR9, leading to the activation of transcription factors such as NF-κB. This promotes pro-IL-1β and pro-IL-18 expressions. Subsequent NLRP3 inflammasome activation results in caspase-1-mediated cleavage of pro-IL-1β and pro-IL-18 into their active forms. IL-1β and IL-18 release exacerbates hepatic inflammation by recruiting neutrophils and monocytes, while inflammasome activation can also induce inflammatory cell death. PAMP: pathogen-associated molecular pattern; PRRs: pattern recognition receptors; TLR4: toll-like receptor 4; TLR9: toll-like receptor 9; pro-IL-1β: pro-interleukin-1beta; pro-IL-18: pro-interleukin-18.

In summary, intestinal flora imbalance can cause an inflammatory reaction in the liver, insulin resistance, and lipid metabolism disorders. This self-reinforcing cycle of intestinal barrier–microbiome–immune system interaction jointly promotes the occurrence and development of MASLD. Research on relevant biological mechanisms provides a theoretical basis for developing treatment strategies for the intestinal flora and inflammatory pathways (Figure 3).

However, there are still many challenges in this field. The gap between animal and human data, along with microbial specificity among individuals, underscores the necessity for human studies to validate causal relationships [94]. To advance this field, research must further develop human cohort studies to verify these mechanisms, accounting for microbiome heterogeneity when assessing barrier function and immune activation. This will be key to transforming the “barrier–immune” axis from a compelling mechanistic concept into a reliable therapeutic intervention target.

### 3.2. The Metabolic Axis: Microbial Metabolites as Hepatic Messengers

The gut microbiome directly affects host metabolism by a range of metabolites, particularly bile acids (BAs), short-chain fatty acids (SCFAs), and choline compounds, which cooperate to form the central metabolic axis implicated in MASLD development. Although the metabolites produced by the microbiome affect the process of liver lipid synthesis and oxidation, lipid clearance, and glucose metabolism through the gut–liver axis, these metabolites can also interact with inflammatory reactions and metabolic regulation through nuclear receptor pathways. This section systematically reviews the correlation mechanism between microbial metabolite-level disorder and the development of MASLD.

#### 3.2.1. Bile Acids (BAs)

Intestinal bacteria in the colon are primarily responsible for converting primary bile acids to secondary bile acids. For example, the bile salt hydrolase (BSH) expressed by *Lactobacillus* and the 7α-/7β-dehydroxylase activity of *Clostridium scindens* jointly catalyze this transformation process [95,96]. In addition, *Clostridium* and other 7α-dehydroxylating bacteria can produce secondary bile acids—deoxycholic acid (DCA) and lithocholic acid (LCA). At the same time, 7β-hydroxysteroid dehydrogenase (7β-HSDH) expressed by certain bacteria can promote the conversion process of hydrophilic and hydrophobic bile acids, and mediate the conversion of ursodeoxycholic acid (UDCA) to chenodeoxycholic acid (CDCA) [97].

Bile acids can also modulate microbiota composition. For example, 3-sucCA can improve intestinal barrier function and diminish systemic inflammation by increasing *Akkermansia muciniphila*, thus exerting favorable effects on diet-induced MASLD in mouse models [98]. Similarly, 3-acetoDCA, which was discovered as a novel and distinct bile acid, can promote the growth of *Lactobacillus*, showing a mutual regulation of bile acids and the microbial ecosystem [99].

The disruption of bile acid homeostasis—characterized by enhanced production, impaired feedback regulation, and altered processing—is recognized as a central mechanism in the progression from MASLD to MASH. Clinical evidence from human studies indicates that MASLD patients exhibit elevated serum and fecal bile acid levels, which correlate positively with disease severity [100,101]. Consistently, human cohort analyses reveal that increased levels of the bile acid synthesis marker *C4* and decreased expression of the enterokine fibroblast growth factor 19 (FGF19) reflect a breakdown in the physiological feedback loop regulating bile acid synthesis [100]. Complementary data from animal models and translational studies further demonstrate that elevated free fatty acids and reduced hepatic small heterodimer partner (SHP) expression upregulate cholesterol 7α-hydroxylase (CYP7A1) and Na^+^-taurocholate cotransporting polypeptide (NTCP), thereby accelerating bile acid biosynthesis and enterohepatic recirculation [102]. Moreover, advanced profiling in human cohorts indicates that not only the quantity but also the composition of the bile acid pool shifts in relation to the degree of insulin resistance [101]. This integrated evidence from clinical observations, animal models, and computational analyses [102,103,104] collectively underscores the pivotal role of bile acid dysregulation in MASLD pathogenesis.

Bile acid regulation requires the participation of two key receptors, farnesoid X receptor (FXR) and Takeda G protein-coupled receptor 5 (TGR5). FXR, which is mainly activated by primary bile acids, has an important impact on metabolism and inflammation through multiple pathways, including inhibiting bile acid synthesis through the FGF19/CYP7A1 pathway [101], enhancing insulin sensitivity through the phosphoinositide 3-kinase (PI3K)/protein kinase B (PKB/AKT) pathway [105,106], inhibiting de novo lipogenesis (DNL) gluconeogenesis by activating peroxisome proliferator-activated receptor (αPPARα) and AMP-activated protein kinase (AMPK) [105,106], and regulating inflammatory and fibrotic reactions through the mitogen-activated protein kinase (MAPK) and mammalian target of rapamycin (mTOR) pathways [105].

On the other hand, TGR5 is mainly stimulated by secondary bile acids [107]. TGR5 activation increases deiodinase 2 (DIO2) expression, which enhances the activation of thyroxine (T4) to triiodothyronine (T3), thus increasing energy expenditure in brown adipose tissue and skeletal muscle [108]. In addition, TGR5 receptor activation can promote glucagon-like peptide-1 (GLP-1) secretion [109,110]. Despite these specific effects, FXR activation in the gastrointestinal tract can regulate microorganism composition. This regulation enhances the TGR5/GLP-1 pathway through a feedback mechanism, thereby improving liver metabolism and insulin sensitivity [111]. The latest research also shows that the intestinal flora dysbiosis observed in MASLD not only leads to the upregulation of secondary bile acid levels in the ileum but also activates the TGR5 receptor on CD8^+^ T cells and finally activates the mTOR/oxidative phosphorylation (OXPHOS) signaling pathway. These processes cause ileum inflammation, bile acid imbalance, and liver FXR function decline, thus promoting the progression of MASLD [112].

In summary, FXR and TGR5, as key regulatory factors of bile acid metabolism, complement each other, although their mechanisms are different. In addition, the intestinal microbiota plays an important and fundamental regulatory role. Future research efforts should be based on an integral model of the intestinal and hepatic interfaces, advocating for individualization based on the profiling of bile acids and intestinal barrier integrity, followed by intervention based on FXR, TGR5, and their pathways to arrest the progress of MASLD.

#### 3.2.2. Short-Chain Fatty Acids (SCFAs)

SCFAs, specifically acetate (C2), propionate (C3), and butyrate (C4), are by-products of the microbial fermentation of dietary fibers [113]. They are important not only for the energetic maintenance of the intestinal epithelium but for many other cellular processes as well [107]. The role of SCFAs in MASLD is multifaceted and cannot be categorized as uniformly protective or detrimental. Their net effect is determined by a complex interplay between dietary composition, host metabolic status, and the functional capacity of the gut microbiota.

The interplay between SCFAs, dietary factors, and the intestinal microbiota is highly dynamic. An enhanced *Firmicutes*/*Bacteroidetes* (F/B) ratio, which characterizes MASLD, is often evidenced in high-fat-induced MASLD murine models. Fermentable fibers like inulin and fructooligosaccharides were proved to increase SCFA production, decrease the F/B ratio, and limit the extraction of dietary energy, thereby mitigating MASLD [114]. Significantly, the disruption of the intestinal microbiota, which characterizes MASLD, could be related to age, as shown. Adult MASLD patients typically have low *Bacteroidetes*, whereas the opposite is true for children [115]. In vivo comparisons involving murine models have shown varying effects of different β-glucans on SCFA production: oat β-glucans were particularly potent in increasing the production of C2 and C3 and in enhancing SCFA-producing microbial populations. This study hinted that the prebiotic activity of fibers varies depending on their origin [116].

SCFAs play important roles in the pathophysiology of MASLD, and their mechanisms are wide-ranging, often synergistic, and sometimes counteracting. Regarding the metabolic aspect, C2 activates the AMPK pathway, which decreases the secretion of chylomicrons and enhanced lipid metabolism [117]. Activation of free fatty acid receptor 2 (FFAR2) by C2 or C3 induces the secretion of gastrointestinal hormones like GLP-1, Peptide YY (PYY), and serotonin (5-HT) [118]. Regarding the functional aspect, the metabolic impact of SCFAs as an energy source is context-dependent. Under conditions of a balanced, high-fiber diet, SCFAs support beneficial functions, including hepatic health and barrier maintenance. Conversely, in the context of a chronic high-fat, low-fiber diet—a hallmark of MASLD—the caloric contribution from SCFAs may add to the overall energy surplus and thus potentially favor metabolic disease progression [119,120].

C3 and C4 increase the intestinal process of gluconeogenesis, which leads to glucose that is detected by the glucose transporter 2 (GLUT2) receptor of the portal vein, thus initiating nerve pathways that decrease food intake and increase the sensitivity of the liver to insulin [121,122]. Immunologically, SCFAs exert an anti-inflammatory effect through reducing inflammation by modulating macrophage polarization, suppressing the infiltration of neutrophils and oxidative damage, and inducing the apoptosis of reactive immune cells. They help B cells mature, enhance mucosal immunity, and reduce the production of autoantibodies. On the other hand, their potential immunologic effect of enhancing Th1 and Th17 effector cell responses, particularly during certain conditions, underscores their complex regulatory role. They induce differentiation of regulatory T cells mediated through inhibition of histone deacetylase (HDAC) and engagement of the FFAR2 and free fatty acid receptor 3 (FFAR3) receptor pathways [123]. The various functions above render SCFAs potential therapeutic targets for the development of personalized treatments that could address the metabolic and immunologic disruptions of MASLD.

#### 3.2.3. Choline, TMA, and TMAO

Choline is an important nutrient and cellular component of cell membranes. It can be obtained from the diet, e.g., from red meat and eggs, or it can be produced endogenously [13]. Recent findings suggest that an imbalance in choline metabolism, which is catalyzed by the presence of the intestinal microbiota, plays an important role in MASLD development and progression [124]. Bacterial species, specifically members of the *Proteobacteria* and *Firmicutes*, catalyze choline into trimethylamine (TMA) through the activity of choline TMA-lyase (CutC/D). TMA passes into the liver through the hepatic portal circulation, where it is oxidatively converted by flavin monooxygenase 3 (FMO3) to trimethylamine N-oxide (TMAO).

This mechanism can be understood from animal research as well, where it was observed that interleukin-33 (IL-33) disruption in mouse models led to an improvement in MASLD symptoms and a decrease in the levels of choline-metabolizing bacteria, including *Lachnoclostridium*, *Desulfovibrio*, and *Prevotella*. This reduction would translate into decreased levels of TMA and TMAO in the serum. Crucially, the levels of their precursor, choline, as well as those of L-carnitine, remained unaltered. This would prove that the observed metabolite levels are the result of microbial enzymatic activity instead of substrate limitation [76], suggesting that the reduction in specific choline-metabolizing bacteria mediated through the disruption of IL-33 protects against MASLD.

The pathway by which gut microbiota converts dietary choline into TMAO is considered one of the potential mechanism axes linking diet, microbiota, and MASLD. In animal models, choline deficiency or increased TMAO production driven by specific microbiota has been demonstrated to exacerbate hepatic steatosis and inflammation through mechanisms including suppression of FXR signaling, induction of mitochondrial dysfunction, and macrophage pyroptosis [125,126,127]. These studies provide crucial mechanistic insights into the causal chain linking choline, TMAO, and liver injury.

However, we must exercise caution when directly extrapolating this mechanism axis derived from animal studies to human MASLD. Although clinical research suggests that serum TMAO levels in MASLD patients correlate with disease severity, the causal relationship remains controversial [128]. Choline may not directly trigger MASLD but rather promote TMAO production against a backdrop of gut dysbiosis, thereby exacerbating existing hepatic inflammatory responses [129]. Choline exhibits bidirectional effects: in the general population, it may exert protective benefits by maintaining hepatic phospholipid metabolism; conversely, in MASLD patients with dysbiosis, choline is more readily converted into the pro-inflammatory mediator TMAO [130]. Furthermore, interindividual variations in microbiota composition significantly contribute to inconsistent research findings: for instance, choline intake correlates with increased stroke risk only in individuals harboring high TMA-producing bacteria [131]. Methodological limitations also warrant attention: existing human observational studies struggle to fully control for the effects of microbiota composition, host genetics, and other dietary variables [129,130,131]. This conflicting evidence suggests that TMAO may reflect host–microbiota co-metabolic states and overall metabolic dysfunction rather than act as an absolute independent pathogenic factor.

Thus, inconsistencies in the current research precisely reveal the highly context-dependent nature of choline metabolism. Future studies require the systematic elucidation of individualized “diet–microbiota–host” interaction patterns in finely stratified population cohorts, integrating metagenomic and metabolomic technologies. This approach will provide the foundation for precision nutritional interventions targeting MASLD.

#### 3.2.4. Other Metabolites

Intestinal microbiota imbalance has been shown to be significantly associated with the generation of endogenous ethanol, an increasingly accepted contributor to MASLD pathology. Studies conducted on patients show a marked presence of ethanol-producing bacteria from *Proteobacteria* and particularly members of the family *Enterobacteriaceae*, as compared to a normal intestinal flora [132]. Their levels show a direct relationship with high concentrations of ethanol present in the portal venous system, which can reach 187 times the normal levels [133]. At a mechanism level, ethanol produced by microbial fermentation may destroy the integrity of the intestinal barrier, causing endotoxins to enter the downstream circulatory system; it can then trigger an inflammatory cascading reaction through the TLR pathway [134]. In addition, after ethanol circulates into the liver through the portal vein, it not only directly produces hepatotoxicity but also continues to stimulate the expression level of metabolic enzymes such as cytochrome P450 2E1 (CYP2E1). These enzymes catalyze the metabolic process of ethanol and produce oxidative stress reactions [135].

Aside from ethanol, the protein-fermenting activity of intestinal microbiota has both harmful and beneficial roles in MASLD. Branched-chain short-chain fatty acids (BCFAs) such as isovalerate and isobutyrate, which are metabolites of microbially broken-down protein, can impair the function of the hepatic tricarboxylic acid cycle, thus leading to steatosis [136]. In contrast, the indole compounds derived from the tryptophan metabolism of intestinal microbiota, such as indole-3-acetic acid (IAA) produced by *Bifidobacterium* species, were shown to be protective against the disease. These compounds decrease the circulating levels of endotoxins, block the activation of macrophages, interrupt the NF-κB pathway, and decrease hepatic inflammation and steatosis [137]. An imbalance between the two groups of metabolites appears to play an important role in the disease.

While microbial metabolites play important roles in the regulation of insulin sensitivity and liver function, most metabolomics analyses are performed after peripheral blood sampling, which would not necessarily provide an ideal representation of hepatic exposure. In a key publication in *Cell Metabolism*, Muñoz and coworkers compared metabolite levels in the portal and peripheral circulation of various mouse models that differed by diet and genetic background. They found that portal vein blood was specifically enriched with metabolites from the gastrointestinal tract. These metabolites activate PPARα, which can enhance fatty acid oxidation and improve mitochondrial function to regulate insulin sensitivity in the liver [138]. This study reveals an important mechanism for direct biochemical communication between the intestine and the liver through microbial metabolites enriched in the portal vein and provides new ideas for metabolic pathway research for the future development of MASLD therapy.

## 4. Therapeutic Strategies Targeting the Gut–Liver Axis

At present, MASLD management revolves around lifestyle measures, specifically diet and exercise. Pharmacologic therapy, such as the use of pioglitazone, vitamins E and D, and statins, exists mainly for the management of complications. However, the use of these measures is limited by the variability in their efficacy and safety [139]. In this context, a pragmatic precision framework may involve (i) microbiome or metabolite profiling, (ii) identification of dominant mechanistic axes (e.g., bile acid dysregulation or barrier dysfunction), (iii) targeted microbiome-modulating interventions, and (iv) evaluation using non-invasive metabolic or imaging-based endpoints.

**Figure 4:**
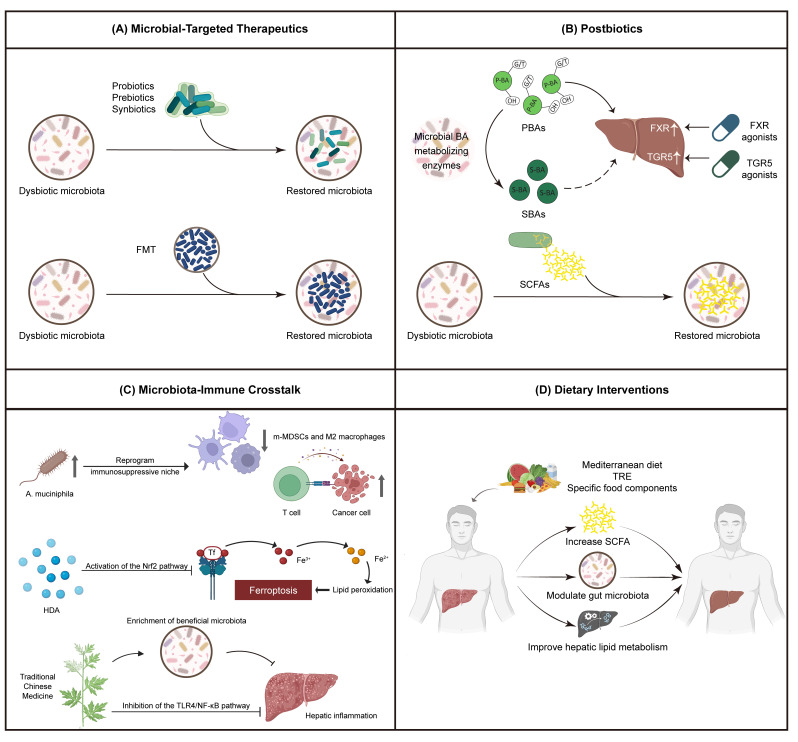
Microbial-targeted intervention measures for MASLD. This figure clarifies the multidimensional treatment strategy of the targeted regulation of the intestinal microbiome and its interactions to treat MASLD. (**A**) Microbial-targeted therapy: This image shows the first generation of microbiome therapy, including probiotics, prebiotics, and synbiotics. This therapy can restore a healthy intestinal flora. Among them, FMT is used to rebuild microbial ecosystems. (**B**) Postbiotics: Postbiotics target microbial metabolic pathways, e.g., via bile acid receptor (FXR, TGR5) agonists or by increasing the level of beneficial metabolites (such as short-chain fatty acids) produced by symbiotic bacteria. (**C**) Bacterial flora–immune interaction: This image emphasizes the immunomodulatory effect of specific microorganisms and their metabolites. Metabolites such as HDA can activate the Nrf2 pathway to inhibit ferroptosis, while traditional Chinese medicine components can inhibit the TLR4/NF-κB pathway to reduce inflammation. (**D**) Dietary intervention: This image introduces dietary strategies such as the Mediterranean diet and TRE. These strategies can adjust the composition and function of the intestinal flora. FMT: fecal microbiota transplantation; HDA: hexadecanedioic acid; Nrf2: nuclear factor erythroid 2–related factor 2; TRE: time-restricted eating.

As knowledge of the pivotal involvement of the intestinal microbiota in the development of MASLD continues to evolve, the use of the intestinal microbiota as an intervention target has come into focus as an innovative area of therapy, which can, to some degree, overcome the existing limitations. Importantly, the translational value of these strategies increasingly lies in their compatibility with non-invasive clinical endpoints, including imaging-based steatosis assessment and circulating metabolite profiles, which now dominate MASLD clinical decision-making [140,141]. This review particularly assesses the potential of the current most advanced microbiota-related treatments based on four major sets of therapy (Figure 4).

### 4.1. Microbiome-Targeted Therapies (MTT)

MTT provides an innovative way to treat MASLD by regulating the intestinal flora. The therapy mainly covers the following interventions: probiotics, prebiotics, synbiotics, and fecal microbiota transplantation (FMT) [142].

The initial microbial-targeted therapy, commonly known as the first-generation therapy, mainly relied on three types of intervention methods: probiotics; prebiotics; and a combination of the two, synbiotics. Clinical evidence indicates that specific probiotic strains (e.g., *Lactobacillus acidophilus* La5, *Lactobacillus casei* TMC, and *Bifidobacterium lactis* Bb12) may improve lipid metabolism, possibly via anti-inflammatory, barrier-enhancing, and antimicrobial mechanisms [143]. In a 10-week trial of mild hypercholesterolemic adults, a milk-based formula (2 × 10^6^ CFU/g per strain) significantly reduced total cholesterol and low-density lipoprotein cholesterol (LDL-c) while also improving intestinal transit. However, direct extrapolation to MASLD is limited by strain specificity, undefined dosing in liver disease, reliance on lipid-based (rather than hepatic histopathological) endpoints, and uncertain long-term safety and feasibility in MASLD populations. Therefore, probiotic applications in MASLD require validation through trials that employ standardized strains, optimized dosing, and liver-relevant clinical outcomes. The new generation of probiotic *Akkermansia muciniphila*, especially the membrane protein Amuc_1100, has shown potential benefits for metabolism despite being pasteurized, thus allowing for the creation of safe and effective products [144]. As far as prebiotics are concerned, there are potential therapeutic effects of inulin-type fructans that selectively increase *Bifidobacterium* and improve insulin sensitivity [145]. Though prebiotics are safe and available, gastrointestinal side effects are possible, as is the potential for liver toxicity [144]. The major potential benefit of synbiotics lies in their theoretical additive and/or synergistic effects, but there are no substantial clinical-data-supported advantages of synbiotics over the other treatments.

FMT is an emerging, novel, and increasingly understood treatment approach that works based on the restoration of a healthier and well-balanced community of intestinal microorganisms. Current evidence demonstrates that the efficacy of FMT in treating MASLD is both time-dependent and population-specific. FMT can improve insulin sensitivity and hepatic fat deposition in the short term (e.g., 6 weeks), but these effects are often transient and may diminish by 18 weeks [146]. Furthermore, FMT appears more effective in lean MASLD patients than in obese individuals [147]. These findings highlight the need for precise patient stratification (e.g., by BMI) and combination with lifestyle interventions to achieve sustained benefit. Regarding safety, FMT is generally well-tolerated, with only mild gastrointestinal symptoms reported and no serious adverse events documented [148]. Clinically, while FMT alone shows limited long-term effects, combining it with diet and exercise significantly reduces liver fat and inflammatory markers [149]. However, broader clinical adoption requires large-scale, long-term trials to optimize dosing and confirm efficacy [150]. To mitigate risks, strict donor screening (including multi-pathogen stool testing) and standardized delivery methods (e.g., colonoscopy or capsules) are essential to minimize infection and adverse event risks [150].

Although MTTs show promise, there are several challenges for their use as an intervention for MASLD. One major limitation is that their efficacy, especially regarding core histopathological outcomes like fibrosis, remains suboptimal. Most trials published to date are small and with short-term follow-up regarding safety and efficacy. Variability in the kind and dose of the probiotics, as well as the endpoint for each study, makes direct comparisons of the treatments difficult. In future, attention needs to focus on the rational selection and design of microbial strains, the optimization of protocols, and the search for mechanistically informative biomarkers that allow patient stratification. Most importantly, well-powered, carefully designed trials are necessary for proving the potential of MTTs as therapy and for incorporating them into future paradigms of MASLD therapy.

### 4.2. Postbiotics

Microbial-derived metabolites and their downstream signaling pathways, referred to collectively as postbiotics, provide potential for novel MASLD therapy, as they offer compensation for impaired host functions [151]. These metabolites represent an innovative category of disease-modifying agents that are theoretically effective in intervening in the host metabolism.

In this context, the modulation of bile acid signaling pathways has received special attention. Both FXR and TGR5 agonists have shown promise, but their use has remained relegated to preclinical investigations. Obeticholic acid (OCA) and similar acids, which are classical FXR agonists, have shown promise regarding improving liver histopathology and biochemical abnormalities, but their adverse effects, such as lipid abnormalities, itch, and constipation, limit their use [152]. Recent data suggest that FXRα FXRβ isoforms have differing influences on the lipid metabolism pathway of the liver, thus possibly allowing for the development of novel therapeutics that would improve efficacy and mitigate adverse effects [153].

In the case of TGR5, the efficacy of tissue-specific agonists varies. While intestinal and liver-specific agonists such as INT-777 show improvement and reduction in liver damage, there are no measurable hepatoprotective effects of systemically administered compounds, such as RO5527239 [154]. Alternatively, the strategy of bile acid sequestration offers an attractive approach that disrupts enterohepatic recirculation, and thus, impacts lipid and glucose metabolism [155,156]. Apical sodium-dependent bile acid transporters have already been confirmed to be effective for hyperlipidemia, but the same must be proven for the present model of MASLD [157].

SCFAs, especially propionate, have proved to be novel, potent microbiota-derived agents that are able to directly influence important pathophysiological aspects of MASLD. This includes decreased lipid deposition, remodeled visceral adipose tissue, and improved energy balance. Mechanistic research provides compelling preclinical evidence for the regulatory effects of SCFAs on the metabolic network at the gut–liver interface [107,139]. Moreover, it was observed that prebiotic supplements are efficient enough to increase the levels of SCFAs as they promote the growth of beneficial bacteria. For instance, study showed that the theabrownins compound derived from Qingzhuan tea (QTB) significantly upregulated the abundance of SCFA-producing microbe populations and enhanced colon SCFA content, which thus inhibited the development of MASLD [158]. However, the potency of SCFA-based treatments appears to be context-dependent. Although sodium butyrate, for example, exerts favorable metabolic effects on healthy people, it loses efficacy for those with pre-existing metabolic problems [159]. This means that tailored treatments are needed.

Nevertheless, despite their broad potential for MASLD, postbiotics and microbial metabolites face many translational hurdles. These include the presence of individual variability in treatments, the unavailability of standardized doses, and the incompletely investigated nature of the safety aspects. Future research efforts should mainly focus on the mechanistic elucidation of host and microbiome interactions and the design of novel, precision-medicine-based approaches.

### 4.3. Microbiota–Immune Interactions

An ever-growing attention has been focused on the symbiotic relationship between the intestinal microbiome and the immune system of the host as it pertains to the regulatory process involved in the development and progression observed in MASLD. Recent research efforts emphasize the potency of specific microbial species, specifically *Akkermansia muciniphila*, to modulate the immune system present within the liver. More specifically, the findings demonstrate the degradation of immunosuppressed myeloid-derived suppressor cells (MDSCs) and M2 macrophages, as well as the enhancement of the effector capabilities of CD4^+^ and CD8^+^ T lymphocytes [160]. These elements provide an underlying mechanism that would support the comparable administration of microbiome-specific treatments and immune system restimulation therapies, particularly applicable within the setting of MASLD-associated HCC.

In addition to microbial species, some gut-derived metabolites have shown immunomodulatory and hepatoprotective properties. Hexadecanedioic acid (HDA), an active compound derived from *Bacteroides uniforms*, exerts liver-protecting activity by triggering the Nrf2/SLC7A11/GPX4 pathway [161]. For instance, the use of the Chinese medicine compound Danggui Shaoyao San was shown to alleviate liver inflammation by modulating intestinal microbial populations and the TLR4/NF-κB pathway [162,163]. This provides some impetus for an integrative model of therapy that uses the best of both traditional and modern knowledge regarding the microbiome for the management of MASLD.

Looking ahead, there are several challenging areas that deserve exploration. These include the design of delivery platforms for targeted metabolites and combination therapies that leverage the strengths of microbial therapy and immunotherapeutic approaches, as well as the use of multi-omics-based paradigms that would support personalized therapy. Overcoming translational hurdles, such as the use of tissue-specific targeting and addressing variability, will surely be the key. Simultaneously, integrating new tools derived from the field of synthetic biology could provide an impetus for developing precision immunotherapeutic approaches for MASLD therapy.

### 4.4. Dietary Interventions

Intestinal barrier function damage and microbiota dysbiosis are the core links of the pathogenesis of MASLD. A growing body of evidence shows that individualized nutritional interventions can alleviate such abnormalities [164]. The application of nutritional intervention strategies such as the Mediterranean diet and time-restricted eating (TRE), as well as the use of functional food ingredients, has shown the potential to reduce liver steatosis and inflammation by regulating the intestinal flora and its metabolites.

At the core of the Mediterranean diet lies the intake of olive oil, fish, and whole grains, focusing on improving the level of dietary fiber and promoting the proliferation of beneficial bacteria such as *Clostridium leptum* and *Eubacterium rectale*. It is the most fully studied and effective MASLD management model at present. This model plays an important role in improving liver inflammation and insulin resistance symptoms by increasing the production of SCFAs, enhancing intestinal epithelial barrier function, and reducing LPS penetration [165]. Several trials have shown that after an observation period of twelve weeks, there was a significant reduction in liver fats, as measured by CAP values, in MASLD patients following the Mediterranean diet. However, observation periods in most trials only last 6–12 weeks, and there is very limited evidence available regarding the long-term efficacy and sustainability of the process [166].

In addition to nutritional content, the timing of eating the nutrient-laden foods becomes an important consideration. TRE methods that are based on eating according to the circadian clock help modulate the metabolism of both the host and the microbial populations [167]. Alongside such interventions, some functional foods show the capability to modulate the microbiome and provide the desired hepatoprotection. For example, 12 weeks of flaxseed supplement administration has been shown to increase the abundance of beneficial microbe levels and reduce hepatic lipid metabolism [168]. Similar benefits were observed for the use of fermented soybeans, which were shown to induce fatty acid oxidation, modulate the intestinal microbiota, and help decrease hepatic steatosis [169]. In addition, active molecules like walnut green husk polysaccharide (WGHP) were shown to have improved efficacy in alleviating high-fat diet-induced MASLD in animal models by increasing the secretion of SCFAs and modulating the intestinal microbiota [170].

Although the potential of such treatments for the prevention and management of various diseases appears well established, there are some limitations to the present state of research on dietary interventions. In fact, most of the existing studies involve short-term follow-up periods and are often conducted using surrogate endpoints as opposed to histopathologic endpoints, without normalizing for the evaluation of fibrosis degree [9]. To advance current research, there is a pressing need for the integration of multi-omics analyses and mathematical modeling to predict individual susceptibility to nutritional therapies. Furthermore, the potential synergies associated with the combination of nutritional therapy, pharmacologic therapy, and exercise could provide the necessary tools for effective MASLD management.

To provide a concise overview of key clinical studies investigating gut–liver axis-targeted interventions in MASLD populations, a list of representative trials is compiled in Table 1. This summary highlights the diversity of approaches—from traditional probiotics and prebiotics to fecal microbiota transplantation and dietary modifications—as well as the variability in study designs, patient populations, and outcomes. Notably, while some interventions show promising effects on metabolic parameters, hepatic fat reduction, and microbiota modulation, the heterogeneity in study duration, sample sizes, and primary endpoints underscores the need for standardized, well-powered trials to establish robust efficacy and optimize therapeutic protocols.

## 5. Future Perspectives and Emerging Frontiers

The microbiome research environment, as it pertains to MASLD, appears to be evolving conceptually from one that focuses on bacteria to one that acknowledges the intricate, multi-kingdom community that inhabits the gastrointestinal tract. This integrative perspective offers innovative mechanistic and therapeutic hints for the gut–liver interface. Specifically, three research fronts, focused on using bacteriophages, antifungal therapies, and metabolites, represent attractive themes for precision microbial modulation.

### 5.1. Bacteriophage Therapy

Although bacterial communities are the main focus of research on the gut microbiome, the gut virome, especially the phage component, has emerged as an important but largely uncharted factor that influences the disease process. In the advanced stages of MASLD, there are some alterations, such as decreased viral diversity and an excessive proliferation of phages that primarily lyse *Escherichia*, *Enterobacteria*, and *Enterococcus* phages [171].

Despite growing attention to such virome disruptions, there are important outstanding questions. Are such disruptions causal for hepatic pathology, or are they sequelae, and how do phages exert their effects, especially as they translate into host immunometabolic responses?

The inherent specificity of bacteriophages for their bacterial targets makes the phage system an attractive tool for targeted microbiota editing. Concept validation experiments have already shown the potential of phage mixtures for reducing liver damage in high-alcohol-producing *Klebsiella pneumoniae* (*HiAlc Kpn*), the steatohepatitis-associated pathobiont, without disrupting the commensal microorganism community [172]. These data confirm the potential use of phage therapy for the precision manipulation of the intestinal microbiota.

Nevertheless, there are many translational hurdles that still exist. These include developing phage-resistant bacterial strains [173], induction of anti-phage immune responses in the host [174,175], and uncertainties regarding the optimum dosage, routes, and duration of phage therapy. The realization of the translational potential of phage therapy would therefore require the development of designed phage cocktails and the use of genetically engineered phages that possess superior pharmacological properties, as well as the development of phage–drug delivery modes.

### 5.2. Antifungal Therapies

Although fungi account for only a small percentage of the total intestinal microbiota, their functional impact is now increasingly understood. The intestinal mycobiome plays an important role in disease pathology as a consequence of microbial interaction, immune system modulation, and the release of biologically active metabolites. In the case of MASLD, the involvement of fungal dysbiosis was implied based on the finding of differences in the distribution of the fungal community and an increase in the levels of serum anti-*Candida albicans* IgG [176], an indicator of active disease, leading to hepatic steatosis and inflammation as a result of intestinal *Candida albicans* colonization [177].

Therapeutic approaches directed against the microbiota are now beginning to emerge. The use of the probiotic yeast species *Saccharomyces boulardii* has already been observed to reduce liver steatosis, inflammation, and fibrosis in various mouse model organisms [178,179]. Similarly, various compounds derived from fungi, such as β-glucans from *Aureobasidium pullulans*, have been discovered to possess liver hepatoprotective properties [180].

An important finding is that *Fusarium foetens* represents a constant intestinal symbiont of the mouse. It provides a protective influence against MASLD by means of the metabolite mechanism. The fungal metabolite FF-C1 was shown to directly interact with the host enzyme ceramide synthase 6 (CerS6) and, as such, to influence sphingolipid metabolism and alleviate the resultant steatohepatitis [181]. This result reveals a new fungal and host metabolic pathway. In combination, these findings highlight the challenges and opportunities for extending the range of MASLD treatments. Multi-kingdom microbiome therapies, encompassing phage and fungal metabolomics components, represent uncharted territory for innovative, mechanism-specific treatments for metabolic liver disease.

### 5.3. Engineered Bacterial Therapy

Probiotic agents engineered with genetic elements are an entirely new approach compared to the traditional method and provide unprecedented control over the temporal and spatial distribution of active compounds exerting their effects within the gastrointestinal system. By genetically engineering the targeted bacterial probiotics, it becomes feasible to repurpose the bacteria to produce and secrete their respective peptides and proteins.

This method has already been validated. For example, an engineered interleukin 22 (IL-22)-producing *Lactobacillus reuteri* significantly alleviates steatohepatitis [182]. Furthermore, engineered glucagon-like peptide 1 (GLP-1)-producing *Escherichia coli* Nissle 1917 and *Lactobacillus gasseri* significantly improve blood glucose metabolism and alleviate hepatic steatosis [183,184].

The use of CRISPR-Cas systems opens up the future possibility of genetic editing of the resident microbiota. This would make it feasible for the resident microbial populations to be directed to produce disease-modifying factors, such as the proteins that are deficient during pathological conditions [185,186].

Although there are very hopeful preclinical results, the translation phase is, as of now, only in the infancy stage. The hurdles that it needs to overcome are the lack of optimal dose regimen development, the scalability of the ongoing trials, and the dominance of homogenous study designs that ignore the obvious heterogeneity of the underlying MASH pathology. To overcome such shortcomings, researchers need to develop a precision design that focuses on designing and developing bacterial strains for remedying defined functional impairments through thorough phenotyping.

Together, these new technologies represent the advent of targeted, mechanism-based treatments, as opposed to the broad-spectrum manipulation of the microbiota. The future of microbiome-based therapy for MASLD, therefore, would seem to lie in the use of phage-guided reduction in pathogenic populations, followed by the use of protective metabolites from fungi, and the use of engineered bacteria for the delivery of curative proteins. This, of course, would necessitate the close study of the individualized microbiome and the use of multi-omics information for the purposes of effective stratification and personalized therapy. In other words, microbiome-based therapy serves as an effective means of implementing causal, disease-modifying responses.

### 5.4. Gut–Brain Axis: Mechanistic and Therapeutic Insights for MASLD

Beyond direct gut–liver communication, experimental evidence indicates that microbiota-mediated gut–brain signaling can modulate host metabolic and inflammatory states relevant to MASLD [187,188,189,190]. Specifically, one study has provided direct experimental evidence that gut microbial metabolites engage vagal afferent signaling to the brainstem, thereby defining a mechanistic gut–brain communication axis relevant to systemic metabolic regulation [191]. In addition, defined microbial functions have been shown to generate neuroactive metabolites that directly alter brain activity and behavior, establishing a causal gut-to-brain axis in liver disease models [192]. Microbiota-driven immune signaling has further been demonstrated to affect brain–metabolic crosstalk through cytokine- and barrier-dependent mechanisms [193]. Together, these experimentally defined pathways suggest that microbiome-based interventions may indirectly modify MASLD progression by targeting gut–brain circuits. Although not yet disease-specific, transcutaneous vagus nerve stimulation has been shown to modulate gastric motility in healthy volunteers [194], indicating the feasibility of targeting gut–brain axis functions in human physiology.

## 6. Conclusions

The gut–liver axis represents an integrative nodal point for the intricate relationship between host genetics, the environment, and the intestinal microbiome in MASLD development. It would be fruitful to reinterpret the relationship between the intestinal microbiome and the development of MASLD. This relationship would no longer be one of simple imbalance but would become one of functional disruption influencing hepatic lipid processing. This review synthesizes current evidence to propose an integrated perspective on MASLD pathogenesis, highlighting how host factors shape gut microbiome functionality along three interrelated axes.

Although many advances have been made, the community stands at a critical inflection point. There is now an urgent need to shift the focus from association-centric paradigms to the development of causality-informed, biological models founded on human biology. Future interventions should shift from generalized supplement approaches to developing new therapies that can manipulate individual functions of microorganisms, and trends need to shift beyond merely focusing on bacterial components.

In order for such an agenda to progress, multi-omics approaches need to be integrated for the purposes of patient phenotyping and stratification, and combination therapies targeting ecological, functional, and immunometabolic aspects of the disease need to be designed. At the same time, the utilization of novel technologies such as microbial therapy and the use of bacteriophages needs to be examined and assessed.

Overall, the therapeutic strategies explored in this paper span a broad spectrum, from gut microbiota modulation to dietary interventions and emerging technologies. Collectively, these approaches outline a potential pathway for improving MASLD management. We must shift our research focus from identifying correlations to uncovering causal relationships, as well as upgrade interventions from broad-spectrum modulation to mechanism-specific targeting, thereby advancing more personalized therapeutic strategies. However, realizing this vision requires overcoming several critical translational hurdles: precise patient stratification, validation of causal mechanisms in humans, and demonstration of long-term efficacy and safety. Future research should integrate multi-omics data and employ innovative trial designs, which are essential for exploring the feasibility of treating MASLD through modulation of the gut–liver axis.

## Figures and Tables

**Table 1:** Summary of human clinical trials on microbiome-targeted therapies for MASLD.

Category	Intervention	Design	Subjects	Key Outcomes	Reference
Probiotics and Prebiotics					
Probiotics	*L. acidophilus* La5, *L. casei* TMC, *B. lactis* Bb12 milk formula	RCT, double-blind, placebo-controlled (10 weeks)	Mildly hypercholesterolemic adults (*N* = 40) N	Positive: Significantly reduced TC (−8.1%) and LDL-c (−10.4%); improved gut microbiota and transit.	Chiu et al. (2021) [143]
Probiotics (Exploratory)	*Akkermansia muciniphila* (pasteurized)	Open-label, single-arm pilot (2 weeks)	Overweight/obese adults with MetS (*N* = 20, safety cohort)	Positive (preliminary): Well-tolerated; showed trends in improved insulin sensitivity and markers.	Plovier et al. (2017) [144]
Prebiotics	Inulin-type fructans (ITFs, 16 g/day)	RCT, double-blind, placebo-controlled (12 weeks)	Obese women (*N* = 30)	Positive: Modestly improved host metabolism, reduced endotoxemia, and correlation with beneficial microbiota shifts.	Dewulf et al. (2013) [145]
Fecal Microbiota Transplantation (FMT)					
FMT	Heterologous FMT via colonoscopy + enemas	RCT, controlled (1 mo follow-up)	MASLD patients (*N* = 75)	Positive: Reduced hepatic fat; improved microbiota diversity; greater efficacy in lean vs. obese patients.	Xue et al. (2022) [147]
FMT	Single allogenic FMT (lean donor) vs. autologous	RCT, double-blind (6 and 18 weeks)	Obese men with MetS (*N* = 38)	Mixed: Transiently improved insulin sensitivity at 6 weeks (predictable by baseline microbiota); effect not sustained at 18 weeks.	Kootte et al. (2017) [146]
Dietary Interventions					
Diet (Mediterranean)	Mediterranean diet (MedDiet) vs. low-fat diet (LFD)	RCT, parallel-group (12 weeks)	Adults with MASLD (*N* = 39)	Neutral (between groups): No significant difference in intrahepatic lipid reduction. Positive (within group): LFD significantly reduced IHL and HOMA-IR.	George et al. (2022) [166]
Diet (Supplement)	Whole flaxseed powder (30 g/day)	RCT, open-label (12 weeks)	Chinese MASLD patients (*N* = 50)	Positive: Significantly reduced liver fat (MRI-PDFF), body fat, and improved lipid profile and gut microbiota.	Tian et al. (2025) [168]
Other/Exploratory Targets					
Postbiotic (SCFA)	Oral sodium butyrate (4 g/day)	Open-label, pre-post (4 weeks)	Lean (*N* = 9) and MetS (*N* = 10) males	Context-dependent: Improved insulin sensitivity in lean subjects only; no effect in MetS patients.	Bouter et al. (2018) [159]
Clinical Biomarker	Fecal *Akkermansia* abundance	Retrospective cohort	HCC patients on anti-PD1 therapy (*N* = 53)	Positive (correlative): Higher abundance correlated with better immunotherapy response and progression-free survival.	Wu et al. (2025) [160]

TC: total cholesterol; LDL-c: low-density lipoprotein cholesterol; RCT: randomized controlled trial; MetS: metabolic syndrome; ITFs: inulin-type fructans; g/day: grams per day; MASLD: metabolic dysfunction-associated steatotic liver disease; mo: month; MedDiet: Mediterranean Diet; LFD: low-fat diet; IHL: intrahepatic lipid; HOMA-IR: Homeostatic Model Assessment for Insulin Resistance; MRI-PDFF: Magnetic Resonance Imaging–Proton Density Fat Fraction; SCFA: short-chain fatty acid; HCC: hepatocellular carcinoma; PD1: Programmed Cell Death Protein 1.

## Data Availability

No new data were created or analyzed in this study. Data sharing is not applicable to this article.
